# Cerebral Fat Migration After Spine Surgery

**DOI:** 10.5334/jbsr.3993

**Published:** 2025-07-04

**Authors:** Dries Vanhoegaerden, Michiel Herteleer, Philippe Demaerel

**Affiliations:** 1 UZ Leuven, department of Radiology, KU Leuven, Belgium; 2 UZ Leuven, department of Traumatology, KU Leuven, Belgium

**Keywords:** Orthopedic procedures, Sacrum, Bone Marrow, Fractures, Bone, Subarachnoid space, Tomography, X-ray Computed

## Abstract

A 73-year-old patient with a complex sacral fracture underwent stabilising orthopaedic surgery. Postoperative brain CT showed fat droplets in the subarachnoid space and lateral ventricle. These fat droplets migrated into the cerebrospinal fluid by a tear in the sacral thecal sac.

*Teaching point:* Intracranial subarachnoid fat droplets may be observed after spinal surgery.

## Introduction

Dissemination of fat into the cerebrospinal fluid (CSF) is a known finding in spontaneous or surgical rupture of an intracranial dermoid cyst [1]. It has also been reported after cranial surgery or a spinal trauma [2–7].

## Case Report

A 73‑year‑old male was admitted to the emergency department after a fall from the stairs. The patient was known with chronic obstructive pulmonary disorder and with a history of alcohol abuse. He was fully conscious and had slightly decreased blood pressure (75/53 mmHg). Physical examination showed a paresis of the left L5‑S1 nerves with reduced flexion (MRC muscle strength grading 1/5).

**Figure 1:**
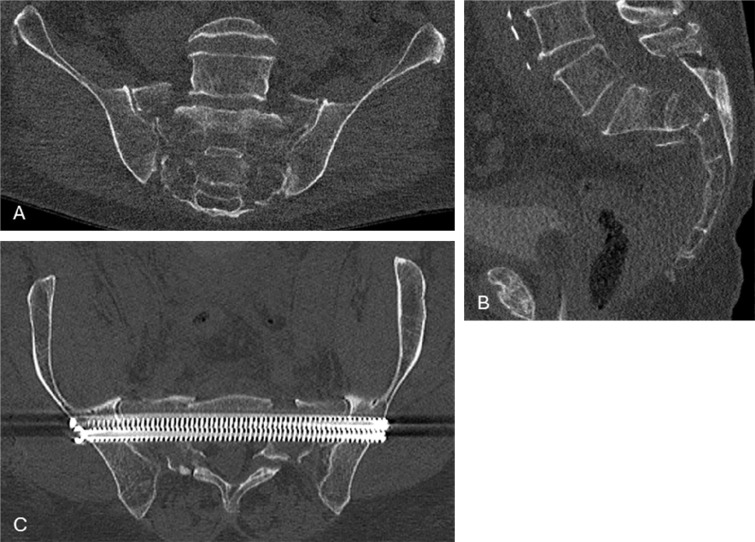
Coronal **(A)** and sagittal **(B)** preoperative images of the U‑shaped sacral fractures. **(C)** The double transsacral–transiliacal screws used for stabilising the fracture.

A full‑body polytrauma CT was performed and showed a displaced U‑shaped pelvic fracture consisting of a transverse sacral fracture with bilateral vertical sacral wing fractures (AO classification C3) as shown in Figure 1. There was some concern about a possible small epidural haemorrhage, but a brain MRI showed no intracranial bleeding. Further findings were unremarkable.

The patient was admitted to the intensive care unit for neurological and haemodynamic observation for 2 days before being transferred to a level I trauma centre for surgery. Two transiliacal–transsacral screws were positioned to stabilise the fracture.

**Figure 2:**
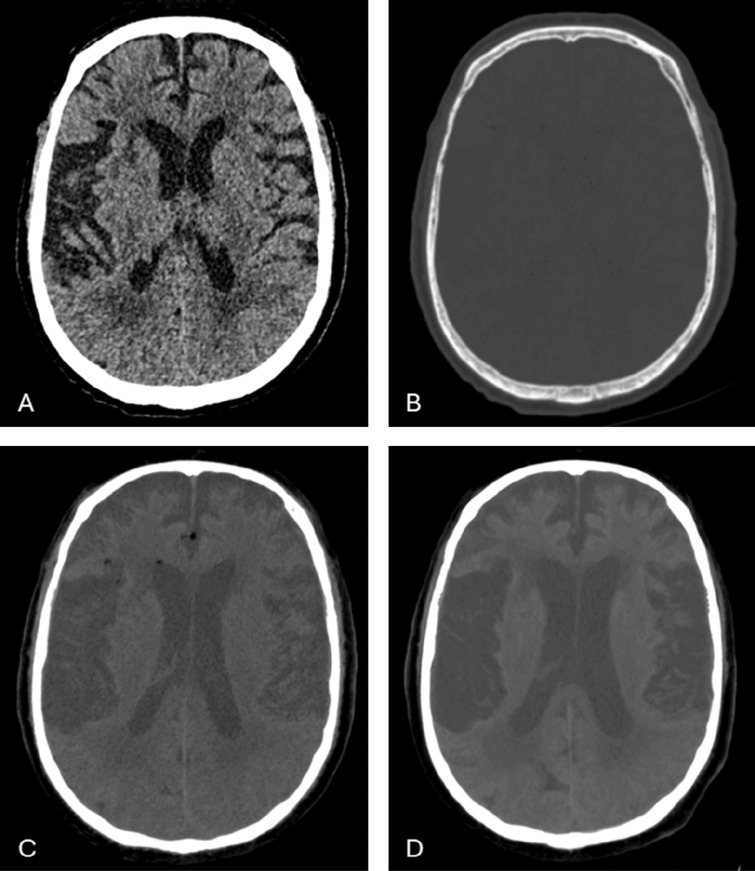
The subarachnoid and intraventricular fat droplets are hardly visible on standard brain window **(A)** and very subtle on bone window **(B)**. 10 mm minimum intensity projection (MinIP) images in the soft tissue window show the true extend of the fat dissemination in the cerebrospinal fluid **(C)**. These were not present on the initial post‑traumatic NCCT **(D)**.

Because of anisocoria the next morning, a brain CT was performed. There was no evidence of recent ischaemia or bleeding, but as shown in Figure 2 several fat droplets were observed in the subarachnoid space and in the lateral ventricles, which were not present on the initial posttraumatic CT.

Since the CT showed no urgent findings, standard follow‑up on the ICU was initiated with spontaneous recovery of the anisocoria.

Because one transsacral–transiliacal screw was in close vicinity with the S1 nerves on both sides, revision surgery was performed by iliosacral screws on both sides.

## Discussion

Trauma involving the head or spine, most often the sacrum, can cause a rupture of the thecal sac and leakage of fatty bone marrow in the CSF [1–7]. Macroscopic intracranial fat in the CSF is usually related to a spontaneous rupture or surgery of a dermoid cyst [7]. When intracranial fat droplets are present after trauma, the retrospective analysis of available previous imaging may exclude pre‑existing fat‑containing lesions. Distant fractures can be associated with a tear in the thecal sac and migration of fat droplets [4–7]. Intracranial fat droplets have been reported after cranial surgery, but this is the first report documenting subarachnoid and intraventricular fat droplets after spinal surgery [2, 3].

Fat in the CSF is asymptomatic, but these patients can show symptoms of spinal fluid leakage or chemical/aseptic meningitis [6, 7].

This finding should be differentiated from cerebral fat embolism that is symptomatic and can be seen after long bone fracture and typically appears as ‘star field pattern’ on brain MRI, reflecting multiple embolic infarcts. Rarely, fat can lead to a large vessel occlusion and infarction, which has been reported after cardiac surgery [8].

## Conclusion

Intracranial fat following surgery is a rare finding and has until now not been reported after spinal surgery. An important prerequisite to depict the fat droplets on CT is to adjust the window width and the brightness level.
